# Tray Rationalization in Pediatric Day Surgery: A Sustainable Quality Improvement Project

**DOI:** 10.1002/wjs.12530

**Published:** 2025-03-08

**Authors:** Eleanor Ferris, Yara Hazem Zaky, Emmy‐Lou Elder, Søren Kudsk‐Iversen, Kokila Lakhoo

**Affiliations:** ^1^ Department of Pediatric Surgery Nuffield Department of Surgical Sciences Oxford University Hospitals Oxford UK; ^2^ Department of Anaesthesia Oxford University Hospitals Oxford UK

**Keywords:** pediatric surgery, planetary health, quality improvement, surgical tray rationalization, sustainable healthcare

## Abstract

**Background:**

Climate change poses a major threat to human health. The decontamination of used surgical equipment has been identified as a “carbon hotspot” in theaters. Surgical tray rationalization, which removes enough instruments to shrink the tray size, has a significant impact on carbon footprint, providing that there is not an increase in individually wrapped instruments used.

**Methods:**

Using the sustainability in quality improvement framework, we rationalized the surgical tray used for pediatric open herniotomies at the Oxford University Hospitals John Radcliffe site. Data on instrument utilization and individually wrapped instruments were prospectively collected. Our tray redesign had no threshold utilization rate for instrument exclusion and focused on removing enough instruments to reduce the tray size. To calculate impact, we used established data on carbon emissions and financial cost and surveyed staff attitudes toward the redesigned tray.

**Results:**

The tray at baseline included 55 instruments. The tray size was reduced by 50% with the removal of 22 instruments. Following our intervention, the median instrument utilization rate increased from 27% to 74% with no significant increase in individually wrapped instruments. The redesigned tray reduced carbon emissions from 4243 gCO_2_e to 2559 gCO_2_e and reduced financial cost from £48.66 to £29.63 per tray per decontamination cycle, approximating to 383,952 gCO_2_e and £4338.84 saved annually. All surveyed staff members (*n* = 25) agreed that the redesigned tray was easy to prepare and felt positive about the effort to reduce environmental impact.

**Conclusions:**

This quality improvement project shows the impact possible by using an established simple and effective framework that can be replicated by healthcare professionals without a background in planetary health to ensure future surgical tray rationalization efforts that maximize environmental impact.

## Graphical abstract

This quality improvement project demonstrates the use of an established, simple, and effective framework for surgical tray rationalisation. The methodology is split into five stages according to the sustainability in quality improvement framework and focuses on removing enough instruments to shrink the tray size without increasing the number of individually wrapped instruments used. The decontamination of used surgical instruments has been identified as a “carbon hotspot“ in theatres. To maximise the impact on population health, we assess the environmental, financial, and social impact of our surgical tray re‐design.

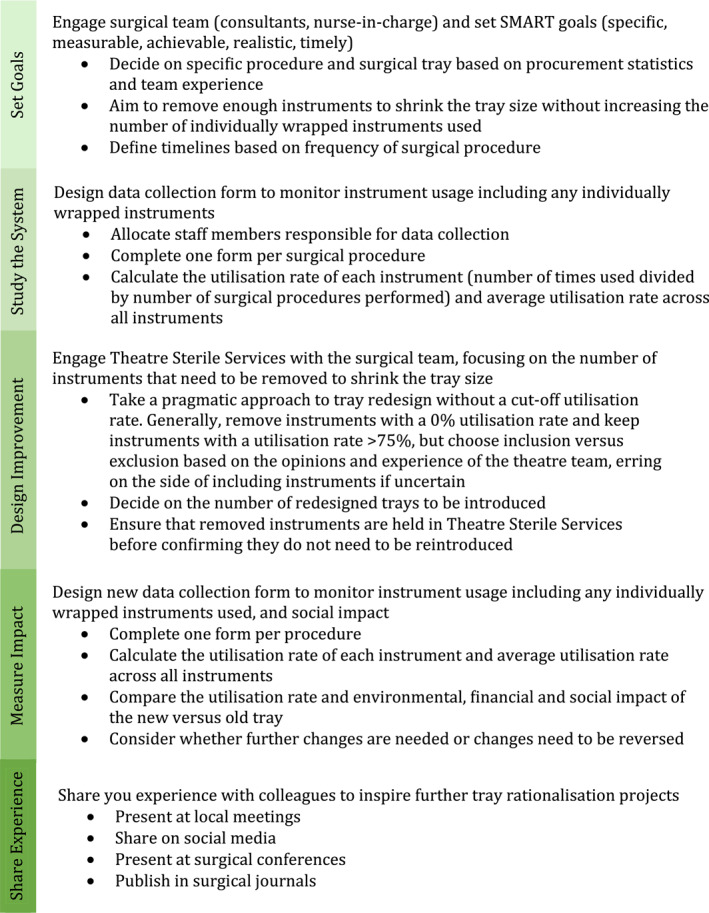

## 1 Introduction

Climate change poses a major threat to human health [1, 2]. Direct and indirect impacts on health outcomes range from heat‐related illness and exacerbations of respiratory disease to increased infections, food insecurity, and mental health issues [2].

NHS England is responsible for an estimated 4% of the country's carbon footprint [3]. In 2020, the NHS became the world's first public health service to commit to reaching carbon net zero for directly controlled emissions by 2040. Setting out a practical evidence‐based path to achieve this, the report “Delivering a Net Zero National Health Service” highlights that the supply chain of medical and nonmedical equipment contributes 18% of the NHS carbon footprint, whereas water and waste contributes 5% [3].

Environmental sustainability has been identified as a critical component in quality improvement (QI) [4]. The sustainable quality improvement (SusQI) framework designed by the Center for Sustainable Healthcare in the United Kingdom encourages clinicians to weigh up the health outcomes for the patient and population against the environmental, social, and financial costs of an intervention–the so‐called “triple bottom line” [4, 5]. In this way, SusQI inspires projects that yield the highest value improvements for both the patients of today and future generations.

Theater is typically the most resource and energy intensive area of a hospital. The carbon footprint of a single operation ranges from 6 to 814 kg of carbon dioxide equivalents (gCO_2_e), with the largest value being equivalent to driving 2273 miles in an average petrol car [6]. The decontamination process, where used surgical instruments are loaded into trays, washed, and sterilized, has been identified as a “carbon hotspot” [6]. Data from four surgical specialties found that over 75% of equipment in 237 instrument trays was not used during the procedure [7], representing an unnecessary and excessive environmental and financial burden.

Surgical trays can be redesigned to minimize the environmental impact of decontamination, but it is critical that this so‐called tray rationalization is informed by quantitative estimates of carbon footprint. For example, the carbon footprint of decontamination can be reduced by simply reducing the number of instruments per tray [8]. However, the greatest impact on carbon footprint comes from reducing the size of the tray since this allows for more trays to fit per washer and sterilizer [8]. Tray rationalization can paradoxically increase carbon emissions if there is an associated increase in the number of individually wrapped instruments used [8]. Therefore, it is important to find a balance between removing enough instruments to shrink the tray size but ensuring that there is not a subsequent increase in the individually wrapped instruments used.

Previous efforts have reduced the number of unused instruments in surgical trays with a focus on financial impact and theater efficiency [9, 10, 11, 12, 13, 14, 15]. A recent effort to reduce unnecessary instruments in a tonsil hemorrhage tray also quantified environmental impact, leading to a 50% reduction in carbon footprint [9]. An Innovation Agency Report published by NHS England identifies key stages of surgical tray rationalization projects, such as engaging stakeholders, data collection, and deciding on an optimum reconciliation strategy [16].

Here, we aimed to rationalize surgical trays used during open pediatric herniotomies with 228 open herniotomies performed in 2023 by 4 full‐time and one part‐time consultant pediatric surgeon/s.

## 2 Material and Methods

**FIGURE 1:**
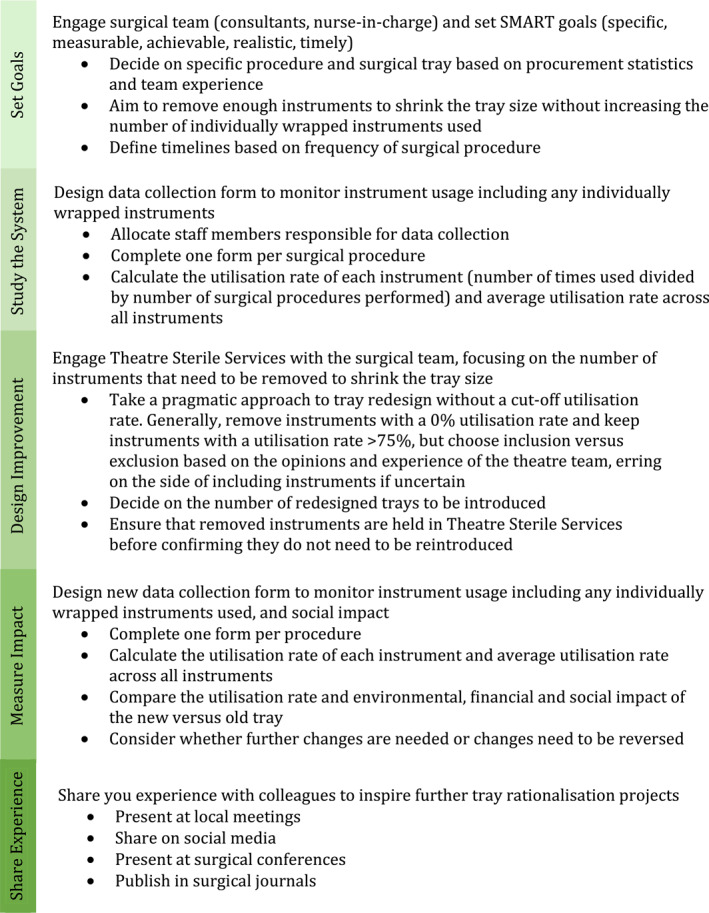
Simplified methodology for a surgical tray rationalization project, split into 5 stages according to the SusQI framework: set goals, study the system, design improvement, measure impact, and share experience. The focus of this method is on removing enough instruments from the tray to reduce its size while ensuring that there is not an increase in individually wrapped instruments used. Environmental, financial, and social impact is assessed. Templates for data collection and staff survey are provided in Supporting Information S1: appendix S1 and S2.

We were guided by the SusQI framework [4]. A summary of our QI methodology is outlined in Figure 1. We report our QI project (QIP) in line with the SQUIRE 2.0 guidelines [17]. Due to the nature of the project, no ethical approval was required, but it was registered and approved by the local QI committee (ID 8864).

*Setting and Population:* Our QIP was conducted in the pediatric surgery department at the John Radcliffe Hospital, part of the Oxford University Hospitals Trust, a tertiary center and teaching hospital, which serves a regional population of 3.5 million from across five counties in the South of England. The department has 4 full‐time and one part‐time consultant general pediatric surgeon/s. Two full‐time consultants perform predominantly open herniotomies and use a laparoscopic approach for very selective cases. The remaining 3 consultants use mainly a laparoscopic approach. The department has 11 surgical trainees and resident doctors and 21 scrub nurses and theater assistants.

**FIGURE 2:**
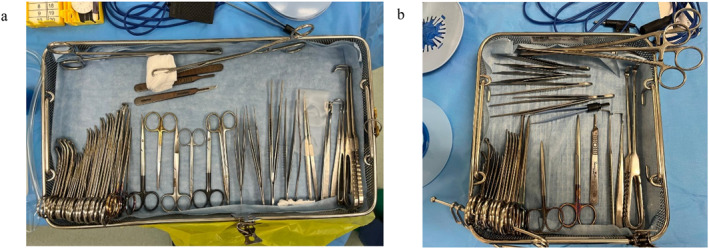
Photographs comparing the 12″ x 24″ aluminum basket preintervention (a) with the 12″ x 12″ aluminum basket after instrument rationalization (b). The redesigned tray is 50% the size of the preintervention tray meaning that two trays fit per decontamination machine slot. The redesigned tray contains 22 fewer surgical instruments.

Each open herniotomy used a “Baby Minor Tray” and soft packed “Pediatric Basic Pack”. Our intervention focused on the “Baby Minor Tray”–a 12″ x 24″ aluminum din basket containing 55 instruments in total (Figure 2a). The “Pediatric Basic Pack” does not contain any surgical instruments but contains a gallipot, kidney dish, drape, and gauze. Each open herniotomy also uses a standard diathermy pencil and blade.

*Data collection:* Baseline data were collected between December 2023 and February 2024 for all open herniotomies using the “Baby Minor Tray,” documenting whether an instrument was used or not (data collection sheet in Supporting Information S1: appendix S1). After tray redesign, similar data were collected for the newly labeled “Baby Hernia Tray” between April and July 2024 (data collection sheet in Supporting Information S1: appendix S2).

*Intervention:* Consensus decision‐making for tray redesign used baseline data and stakeholder inputs. Initially, instruments with a utilization rate of 0% were removed and instruments with a utilization rate above 75% were included. For instruments with a “borderline” utilization rate above 0% but less than or equal to 75%, stakeholder discussions on topics, including when the instruments might be required and variations in surgical practice dictated whether to include or exclude the instrument and verbal agreement of inclusion or exclusion of instruments with a utilization rate above 75% or 0%, respectively, was made.

Stakeholders included the lead and manager of the theater sterile services unit (TSSU), consultant pediatric surgeon (K.L), and the nurse‐in‐charge (E.LE). To avoid service interruption following tray redesign, instruments removed from the original tray were held in TSSU until stakeholders were satisfied that the new tray was satisfactory.

*Analysis and Study of the Intervention:* In line with the SusQI framework, we used prespecified metrics to review the environmental, financial, and social impacts of our intervention [5]. We adopted two methods using established data from a similar setting [8] to calculate environmental and financial impact with an exchange rate of £0.84 to €1. Firstly, we used estimates for individual instruments within trays (77gCO_2_e; £0.88) and individually wrapped instruments (189gCO_2_e; £6.17) multiplied by the number of instruments. Secondly, we used estimates per decontamination machine slot (1531gCO_2_e; £24.87).

We used simple descriptive analysis using median and range as percentages to review utilization rate, carbon dioxide equivalents, and cost per tray per decontamination cycle. A decontamination cycle was defined as a complete cycle through a washer followed by a steam sterilizer both powered by electricity. We used a heat map to visualize instrument usage.

Since we used both utilization rates and stakeholder engagement to decide on content of the new tray, we undertook sensitivity analysis to compare financial and environmental impacts if we had used only preset cutoff utilization rates.

To estimate social impact, we circulated a four‐question survey following introduction of the new tray (survey in Supporting Information S1: appendix S2). Only staff members who prepared or used the tray were eligible for survey completion including consultant pediatric surgeons, surgical trainees, and scrub nurses. Scrub nurses completed the survey on paper at the time of data collection, whereas other members of staff were invited to complete the survey online.

## 3 Results

Data were collected for 24 open herniotomies from December 2023 to February 2024 (preintervention) and 21 open herniotomies from April to July 2024 (post‐intervention). Following a review of instrument usage, we reduced the number of instruments from 55 to 33 (Table 1). With this 40% total instrument reduction, the tray size could be reduced from a 12″ x 24″ aluminum basket to a 12″ x 12″ aluminum basket (Figure 2). This 50% reduction in tray size meant that 12 instead of 6 trays could fit per decontamination machine, leading to a 50% increase in optimal loading efficiency for both the washer and steam sterilizer.

**TABLE 1:** Quantity, average utilization rate, and individually wrapped surgical instruments used pre‐ and post‐ tray redesign.

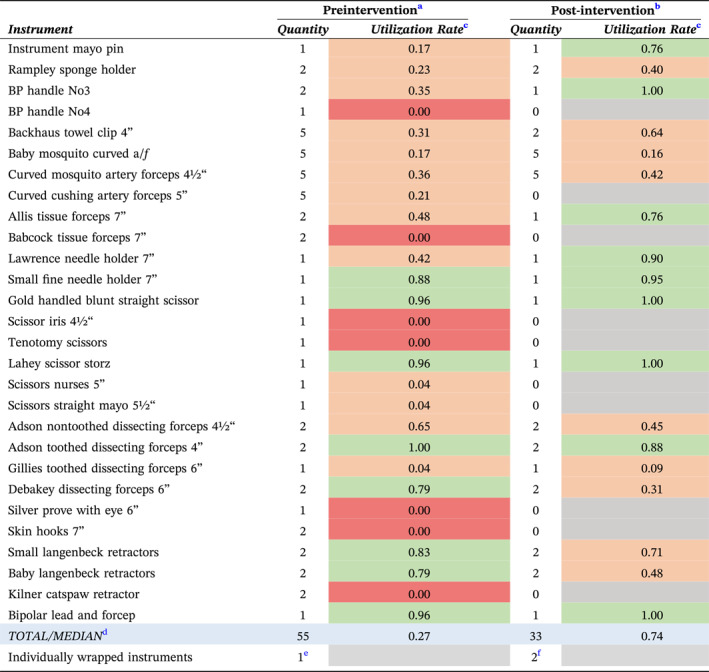

^a^ Utilization rate is calculated as the number of times the instrument is used divided by the number of open herniotomies performed (*n* = 24).

^b^ Utilization rate is calculated as the number of times the instrument is used divided by the number of open herniotomies performed (*n* = 21).

^c^ Instruments with a utilization rate above 75% (green) were considered frequently used; instruments with a utilisztion rate between 0% and 75% (amber) were considered “borderline”; and instruments with a utilization rate of 0% (red) were considered very rarely used.

^d^ Total number of instruments per tray and median utilization rate for the tray preintervation and post‐intervention.

^e^ One individually wrapped instrument (tissue scissors) was used across 24 open herniotomies preintervention.

^f^ Two individually wrapped instruments (nurses scissors and iris scissors) were used across 21 open herniotomies post‐intervention.

The median instrument utilization rate increased from 27% (range 0%–100%) to 74% (range 9.5%–100%) (Table 1). Across 24 open herniotomies preintervention, one individually wrapped instrument was opened compared to 2 individually wrapped instruments post‐intervention.

Using individual instrument estimates, the redesigned tray reduced carbon emissions from 4243gCO_2_e to 2559gCO_2_e (Table 2)–saving the equivalent of driving 4.2 miles in the average passenger vehicle [18] per tray and approximating to 383,952gCO_2_e saved annually. The redesigned tray reduced financial cost from £48.66 to £29.63 per tray per decontamination cycle (Table 2) approximating to £4338.84 saved annually. Using estimates per decontamination machine slot, the redesigned tray reduced carbon emissions from 1531gCO_2_e to 766gCO_2_e and cost from £24.87 to £12.44, fitting two trays per slot instead of one.

**TABLE 2:** Summary of instrument quantity, median instrument utilization rate, carbon footprint, and financial cost pre‐ and post‐ tray redesign.

	Preintervention	Post‐intervention
Open herniotomies performed (*n*)	24	21
Total instruments (*n*)	55	33
Types of instruments (*n*)	28	18
Median utilization rate (%)	27	74
Carbon footprint (gCO_2_e)^a^	4243	2559
Financial cost (£)^b^	48.66	29.63

^a^ Estimates are provided as an average per tray per decontamination cycle including carbon emissions per instrument per set (77gCO_2_e multiplied by number of instruments in the set) and average carbon emissions from individually wrapped instruments (189gCO_2_e multiplied by number of individually wrapped instruments divided by number of open herniotomies performed).

^b^ Estimates are provided as an average per tray per decontamination cycle including cost per instrument per set (£0.88 multiplied by number of instruments in the set) and average carbon emissions from individually wrapped instruments (£6.17 multiplied by number of individually wrapped instruments divided by number of open herniotomies performed).

Sensitivity analysis demonstrated that using consensus decision‐making for tray redesign had a greater impact on carbon footprint and financial cost than using a cutoff utilization rate (Table 3).

**TABLE 3:** Sensitivity analysis demonstrating carbon emissions and financial cost per tray and for individually wrapped instruments if our tray redesign excluded instruments with an average utilization rate of 0%–25%, 0%–50%, or 0%–75%.

	Preintervention	Excluding 0%–75% IUR	Excluding 0%–50% IUR	Excluding 0%–25% IUR	Consensus decision making
Number of instruments in tray (*n*)	55	12	14	29	33
Individually wrapped instruments required (*n*)	1	198	179	52	2
Carbon emissions per tray (gCO_2_e)	4235	924	1078	2233	2541
Carbon emissions from individually wrapped items per tray (gCO_2_e)	8	1782	1611	468	18
Total carbon emissions per tray per decontamination cycle (gCO_2_e)	4243	2706	2689	2701	2559
Cost per tray (£)	48.40	10.56	12.32	25.52	29.04
Cost from individually wrapped instruments per tray (£)	0.26	58.17	52.59	15.28	0.59
Total cost per tray per decontamination cycle (£)	48.66	68.73	64.91	40.80	29.63

Abbreviation: IUR, instrument utilization rate.

Twenty five of 37 (68%) eligible staff completed the post‐intervention survey. 100% of respondents agreed that the redesigned tray was easy to prepare and use. 68% agreed that the redesigned tray improved the efficiency of the theater team in terms of “speeding up counts”, whereas 32% thought there was no change in efficiency. 100% felt positive that the department were making efforts to minimize their environmental impact and welcomed continued use of the redesigned tray for open herniotomies.

## 4 Discussion

We removed 22 instruments from the surgical tray used for pediatric open herniotomies allowing a 50% reduction in tray size. This reduced the number of unused instruments, with an associated reduction in carbon emissions and financial cost from the decontamination process. The redesigned tray fostered positive staff attitudes toward their environmental impact with no subjective detriment to the efficiency of the theater team. This QIP demonstrates the effective use of a simple and replicable framework for tray rationalization projects, which engages the relevant stakeholders and ensures environmental, financial, and social impact is appropriately assessed.

Previous efforts to rationalize surgical instrument trays [9, 10, 11, 12, 13, 14, 15] have focused on reducing perioperative costs [9, 10, 11, 12]. Farrelly et al. calculated an annual instrument cost avoidance which they defined as the theoretical savings that would be gained by avoiding the purchase of the eliminated instrument over 1 year [10]. After removing an average of 39.5% of instruments from 6 general surgical trays, annual instrument cost avoidance was between $9763 and $97,628, with the wide estimated range due to the expected life expectancy of each instrument ranging from 1 to 10 years [10]. Knowles et al. removed 49 instruments in total from a vascular and aortic tray leading to annual savings of $97,444 based on 201 procedures performed over 3 months and an estimated resterilization cost of $1.07 per instrument [11]. Nast et al. reduced the number of instruments in a genitourinary tray by 22 and estimated that cost saved per procedure ranged from $11.22 to $70.18 depending on the study from which estimates were taken [12]. This variation is likely contributed by differences in local operating processes such as detergent used, method of sterilization, and energy source as well as methodological differences such as including or excluding instrument replacement, machinery maintenance, or labor costs in estimates. This highlights the caution needed in extrapolating operating costs from previous studies and limits the generalizability of our findings.

Cost‐effectiveness is critical to maximize health outcomes [19]; however, with the major threat to human health that climate change poses [1, 2], environmental impact should be a nonnegotiable cornerstone of surgical tray rationalization. There has been one published effort that we have found to date which quantifies the environmental impact of tray rationalization [9]. Van Osch et al. reduced the number of instruments from 18 to 8 in a tonsil hemorrhage tray used in the emergency department, and in doing so, repackaged the instruments in a small kidney basin instead of a large tray [9]. This led to an absolute reduction of 32.6gCO_2_e per tray which is significantly less than our estimated absolute reduction of 1684gCO_2_e per tray using individual instrument estimates or 765gCO_2_e using estimates per decontamination machine slot. This is despite Van Osch et al. using the same previously published data to make their estimates of carbon footprint as we used [9]. When we recalculated the estimated carbon impact for the tray redesign by Van Osch et al. using out methods, we found an absolute reduction of 770gCO_2_e per tray per decontamination cycle. To be confident in estimates of carbon emissions moving forward, it is critical that there is methodological clarity in how authors estimate carbon emissions to avoid over or underestimates of environmental impact being made. We used more than one method of calculating environmental impact to demonstrate a range of estimates.

In three previous surgical tray rationalization projects, there was an arbitrary cutoff utilization rate, where instruments used less than 20% of the time were removed [12, 13, 14]. However, in two of these projects, each instrument was also discussed among stakeholders before deciding on the finalized redesigned tray [12, 13]. For Nast et al., this led to the inclusion of S‐retractors despite a 16% utilization rate and the exclusion of Senn retractors despite a 33% utilization rate [12]. For the other four surgical tray rationalization projects, no “cutoff utilization rate” was employed, rather tray redesign was dictated by stakeholder discussions on an instrument‐by‐instrument basis [9, 10, 11, 15]. For example, Farrelly et al. eliminated an instrument if there was a unanimous agreement among surgeons [10]. Knowles et al. made special consideration in stakeholder discussions to instruments with limited use but rapid need if an emergency arose and to high‐usage instruments requiring more than one instrument per tray [11]. In our initial tray redesign, we excluded instruments with a utilization rate of 0% and included instruments with a utilization rate above 75%. Further discussion with lead surgeon, nurse‐in‐charge, and TSSU management finalized the exclusion of 22 instruments. Of these excluded instruments, 12 had a “borderline” utilization rate (above 0% but 75% or less). Our sensitivity analysis (Table 3) was performed to demonstrate the theoretical environmental and financial impact–considering instruments per set and individually wrapped instruments–we had using various cutoff utilization rates for exclusion of instruments in tray redesign. We demonstrated that compared to a 25%, 50%, and 75% cutoff for instrument exclusion, consensus decision‐making led to a greater environmental, and financial impact per tray per decontamination cycle. This also emphasizes the importance of monitoring individually wrapped instrument use given that carbon emissions per individually wrapped instrument is over double an instrument included in a set [8].

The Center for Sustainable Healthcare in the United Kingdom regards social impact as important to identify unintended negative impacts on different groups of people [20]. Staff attitudes were an important aspect of our social impact. For example, tray redesign carried a potential to frustrate or disengage staff members particularly if the tray was more challenging to prepare and use. In addition, given the major health impacts of climate change [1, 2] and the significant carbon footprint of NHS England [3], we wanted to gauge staff attitudes toward tray redesign, which had a positive impact on the carbon footprint of their workplace. Our survey included four questions considering attitudes toward ease of preparation, theater efficiency, environmental impact, and future use of the redesigned tray (survey in Supporting Information S1: appendix S2). Previous tray rationalization efforts have not surveyed staff attitudes [9, 10, 11, 12, 13, 14, 15].

This QIP is not without limitations. For example, there is evidence that loading washers and sterilizers at one‐third capacity increases the carbon footprint by 2.6 times more than typical loading [8]. However, we were unable to identify whether washers and sterilizers were loaded to 100% for decontamination of the redesigned tray. In addition, this is a single‐center QIP utilizing a lifecycle assessment of surgical tray sterilization practices in the United Kingdom. Therefore, caution needs to be taken if looking to replicate in settings outside of the United Kingdom as among other things, energy sources, and decontamination processes may differ.

Addressing environmental challenges as part of QI enables healthcare professionals to safeguard population health from climate change. Here, we combine the SusQI framework with prior insight into effective rationalization strategies to present an approach to surgical tray rationalization. We encourage healthcare professionals with or without a specialist background in planetary health–including medical students, senior trainees, consultants, and scrub nurses–to lead surgical tray rationalization projects locally.

## Author Contributions

**Eleanor Ferris:** conceptualization, data curation, formal analysis, investigation, methodology, project administration, visualization, writing – original draft, writing – review and editing. **Yara Hazem Zaky:** data curation, investigation, project administration. **Emmy‐Lou Elder:** investigation, project administration, Resources. **Søren Kudsk‐Iversen:** conceptualization, methodology, writing – review and editing. **Kokila lakhoo:** conceptualization, methodology, resources, supervision, writing – review and editing.

## Conflicts of Interest

The authors declare no conflicts of interest.

## Supporting information

Supporting Information S1
