# Supplementary material for: Tray Rationalization in Pediatric Day Surgery: A Sustainable Quality Improvement Project
Source: World J Surg. 2025 Mar 8;49(4):1082–9. doi: 10.1002/wjs.12530 (PMC11994143; doi:10.1002/wjs.12530)
Supplement: Supplementary file 1 — Supporting Information S1 [file WJS-49-1082-s001.docx]

*SUPPLEMENTARY MATERAL*

*Title:* Tray Rationalisation in Paediatric Day Surgery: a Sustainable Quality Improvement Project

Eleanor Ferris; Yara Hazem Zaky; Emmy-Lou Elder; Søren Kudsk-Iversen; Kokila Lakhoo

*Corresponding Author:*

Dr Eleanor Ferris

Department of Paediatric Surgery, Nuffield Department of Surgical Sciences, Level 6, Oxford University Hospitals, Headley Way, Oxford, OX3 9DU

[eferris@hotmail.co.uk](mailto:kokila.lakhoo@paediatrics.ox.ac.uk)

+447758911398

APPENDIX S1

The aim of this Quality improvement project is to understand **what instruments/equipment are used** during open paediatric hernia repair and design a repackaging strategy for surgical trays to reduce unused equipment and energy usage in decontamination.

PLEASE COMPLETE ONE FORM PER OPEN HERNIA REPAIR

*Date form completed:*

Please write the quantity used during the surgery

| ***Instrument*** | ***Quantity in tray*** | ***Quantity used*** |
| --- | --- | --- |
| Instrument mayo pin | 1 |  |
| Rampley sponge holder 9” | 2 |  |
| BP handle no3 | 2 |  |
| BP handle no4 | 1 |  |
| Backhaus towel clip 4” | 5 |  |
| Baby mosquito curved a/f | 5 |  |
| Curved mosquito artery forceps 4.5” | 5 |  |
| Curved cushing artery forceps 5” | 5 |  |
| Allis tissue forceps 7" | 2 |  |
| Babcock tissue forceps 7” | 2 |  |
| Lawrence needle holder 7" | 1 |  |
| Small fine needle holder | 1 |  |
| Gold handled blunt straight scissor | 1 |  |
| Scissor Iris 4.5” | 1 |  |
| Tenotomy scissor storz | 1 |  |
| Lahey scissor storz | 1 |  |
| Scissors nurses 5” | 1 |  |
| Scissor straight mayo 5.5” | 1 |  |
| Adson non toothed dissecting forceps 4.5” | 2 |  |
| Adson toothed dissecting forceps 4" | 2 |  |
| Gillies toothed dissecting forceps 6" | 1 |  |
| 6" debakey dissecting forcep | 2 |  |
| Silver probe with eye 6” | 1 |  |
| Skin hooks 7” | 2 |  |
| Small langenbeck retractors | 2 |  |
| Baby langenbeck retractor | 2 |  |
| Kilner catspaw retractor | 2 |  |
| Bipolar lead & forcep | 1 |  |

**ADDITIONAL INSTRUMENTS / EQUIPMENT**

Please list any additional instruments used during the surgery

| Instrument | Quantity |
| --- | --- |
|  |  |
|  |  |
|  |  |

APPENDIX S2

The aim of this QI project is to design a new tray for paediatric open hernia repair which reduces the number of unused surgical instruments. This is a re-audit for the redesigned “baby minor” tray which is smaller with fewer surgical instruments.

PLEASE COMPLETE ONE FORM PER OPEN HERNIA REPAIR

1. Date form completed:
2. I am completing this form for an **open hernia repair** using the **redesigned (smaller)** “baby hernia tray”

Please tick here to confirm

1. For each instrument in the redesigned “baby hernia tray” please write the **quantity used during the surgery**. If the instrument was not used at all, please write “0”

| ***Instrument*** | ***Quantity in tray*** | ***Quantity used*** |
| --- | --- | --- |
| Instrument mayo pin | 1 |  |
| Rampley sponge holder 9” | 2 |  |
| BP handle no3 | 1 |  |
| Backhaus towel clip 4” | 2 |  |
| Baby mosquito curved a/f | 5 |  |
| Curved mosquito artery forcep 4.5” | 5 |  |
| Allis tissue forceps 7" | 1 |  |
| Lawrence needle holder 7" | 1 |  |
| Small fine needle holder | 1 |  |
| Gold handled blunt straight scissor | 1 |  |
| Lahey scissor storz | 1 |  |
| Adson non toothed dissecting forceps 4.5” | 2 |  |
| Adson toothed dissecting forceps 4" | 2 |  |
| Gillies toothed dissecting forceps 6" | 1 |  |
| 6" debakey dissecting forcep | 2 |  |
| Small langenbeck retractors | 2 |  |
| Baby langenbeck retractor | 2 |  |
| Bipolar lead & forcep | 1 |  |

1. Please list any additional individually wrapped instruments that are used during the surgery.

| ***Individually wrapped instrument*** | ***Quantity*** |
| --- | --- |
|  |  |
|  |  |
|  |  |
|  |  |

1. Please complete the multiple-choice questions below

The redesigned tray was easy to prepare and use [in particular, asking the scrub nurse]

Agree

Disagree

The redesigned tray improved the efficiency of the theatre team

Agree

No change

Disagree

I feel positive that the department are making efforts to minimise their environmental impact

Agree

Disagree

I would welcome continued use of this tray for open hernia repairs [asking the whole team]

Agree

Disagree

1. Do you have any additional comments on the redesigned “baby hernia tray”?
